# Microclimate characteristics, temporal dynamics of soil bacterial communities, and potential co-occurrence network differences between greenhouse and open-field cultivation environments

**DOI:** 10.3389/fmicb.2026.1988234

**Published:** 2026-10-16

**Authors:** Lirong Xue, Yonglin Chen, Yihui Zhong, Chenglai Wan

**Affiliations:** School of Geography and Environmental Engineering, Gannan Normal University, Ganzhou, Jiangxi, China

**Keywords:** co-occurrence network, microclimate, soil bacterial community, soil thermal-moisture dynamics, temporal dynamics

## Abstract

**Introduction:**

Greenhouse cultivation modifies agricultural microclimates and may be associated with temporal changes in soil microbial communities. We investigated a representative vegetable greenhouse and an adjacent open-field site in the subtropical red-soil region of Jiangxi Province, China.

**Methods:**

Air temperature and relative humidity were monitored continuously for one year in both environments, while soil temperature and volumetric water content were monitored within the greenhouse. Monthly soil samples were analyzed using 16S rRNA gene amplicon sequencing.

**Results:**

The greenhouse exhibited pronounced daytime warming, nighttime heat retention, and generally higher relative humidity. Soil bacterial community composition differed significantly between greenhouse and open-field soils, while alpha-diversity differences varied among seasons. Month-resolved analyses revealed significant temporal dynamics. Air temperature and relative humidity jointly explained 15.59% of the variation in bacterial community structure. Potential co-occurrence networks also differed between the two environments in topology and in the proportions of positive and negative correlations.

**Discussion:**

The results document year-round differences in microclimate and soil bacterial community dynamics between greenhouse and open-field cultivation environments. The measured atmospheric microclimate variables showed statistically detectable but limited associations with bacterial community composition, providing a basis for soil microbiome monitoring and environmental management in subtropical protected agriculture.

## 1 Introduction

Protected agriculture extends crop production seasons, improves land-use efficiency, and supports year-round vegetable supply through artificial covering and environmental regulation, and has therefore become an important component of intensive agricultural production (Li et al., 2022; Bon et al., 2026). Compared with open-field cultivation, greenhouse production creates a relatively enclosed environment and is accompanied by intensive management practices such as irrigation, fertilization, ventilation, and heat conservation, thereby modifying both the atmospheric microclimate experienced by crops and the soil environment. Long-term greenhouse cultivation and intensive water and fertilizer inputs can also alter soil nutrient status and coincide with shifts in bacterial community composition and assembly processes (Zhang et al., 2024a; Zhang et al., 2024b). Long-term greenhouse cultivation has likewise been associated with substantial changes in microbial community composition and its relationships with ecosystem functions (Xue et al., 2020). Thus, greenhouse agriculture represents not only a crop-production facility but also a distinct agricultural land-use system characterized by specific microclimatic and management conditions that may exert persistent effects on soil ecological processes.

Greenhouse covering primarily modifies energy and water exchange within the crop-growing environment. Covering structures, solar radiation, ventilation, and crop transpiration jointly shape the greenhouse microclimate (Rabbi et al., 2019), and recent measurements across multi-span greenhouses have shown that ventilation conditions and crop canopy height substantially influence the distributions of temperature and relative humidity (Mao et al., 2024). Greenhouse soil temperature also exhibits pronounced temporal and vertical variation and is jointly affected by greenhouse structure and external meteorological conditions (Jiao et al., 2024). Previous studies further indicate that land-atmosphere interactions differ between greenhouse and outdoor environments because covering modifies near-surface energy and water exchange. Continuous monitoring of both atmospheric microclimate and soil thermal-moisture dynamics within greenhouses can therefore improve understanding of the temporal characteristics and potential ecological implications of protected cultivation environments.

Soil microorganisms are highly responsive to changes in temperature, moisture, nutrient supply, and agricultural management. Controlled experiments have shown that changes in moisture and nitrogen inputs can rapidly restructure bacterial community composition and network connectivity (Han et al., 2023), while warming responses may depend on crop type (Zhong et al., 2024). During long-term greenhouse tomato monoculture, rhizosphere bacterial communities can shift together with soil properties, with nitrogen and phosphorus availability closely associated with community changes and with concurrent changes in co-occurrence network stability (Dang et al., 2023). Long-term intensive greenhouse cultivation has also been associated with changes in bacterial community composition and assembly processes and with reduced co-occurrence network complexity (Zhang et al., 2024a). Likewise, long-term drip irrigation can alter salinity and nutrient conditions together with microbial community structure and potential associations (Wang et al., 2023), and different saline irrigation regimes can modify soil physicochemical properties and bacterial community structure (Guo et al., 2023). Co-occurrence network analysis can therefore complement diversity and taxonomic analyses by describing differences in potential microbial association structures among cultivation environments. However, such networks represent statistical co-variation and should not be interpreted as direct evidence of ecological interactions.

Although previous studies have documented relationships among greenhouse cultivation, soil physicochemical properties, microbial community composition, and co-occurrence networks, many have focused on cultivation duration, management treatments, or comparisons at individual time points. Studies that combine year-round microclimate monitoring of a greenhouse and an adjacent open field with monthly analyses of soil bacterial communities remain relatively limited. This knowledge gap is particularly relevant in subtropical red-soil vegetable-production regions, where the seasonal dynamics of atmospheric microclimate, greenhouse soil thermal-moisture conditions, and month-to-month bacterial community variation have not been systematically characterized together. Moreover, microbial communities are simultaneously influenced by soil physicochemical properties, crop identity, irrigation, fertilization, and other management practices; consequently, statistical associations between microclimate and microbial communities must be distinguished from causal environmental drivers.

Accordingly, we conducted a one-year observational study in a representative greenhouse and an adjacent open-field vegetable-production system in a subtropical red-soil region. Our objectives were to: (1) compare year-round air temperature and relative humidity dynamics between greenhouse and open-field environments and describe temporal variation in greenhouse soil temperature and soil volumetric water content (VWC); (2) characterize differences in soil bacterial alpha diversity, community composition, and month-to-month dynamics between the two environments; (3) compare the topology of potential bacterial co-occurrence networks; and (4) evaluate statistical associations between the measured atmospheric microclimate variables and bacterial community structure. By resolving year-round temporal dynamics in microclimate and soil bacterial communities, this study provides ecological information relevant to environmental regulation, soil microbiome monitoring, and water and fertilizer management in subtropical protected agriculture.

## 2 Materials and methods

### 2.1 Study site, soil sampling, and environmental monitoring

The study was conducted in Liangcun Town, Xingguo County, Ganzhou, Jiangxi Province, China (26.55° N, 115.58° E). The study area is located in the hilly region of southeastern China and has a subtropical monsoon climate. The study system comprised an intensively managed plastic greenhouse with approximately 12 years of continuous vegetable cultivation and a spatially adjacent open-field vegetable plot. Both sites were managed according to local conventional vegetable-production practices and were used to compare soil bacterial community composition and temporal dynamics under contrasting cultivation environments.

Seasonal crop replacement occurred in both environments during the monitoring period from October 2024 to September 2025. From October 2024 through March 2025, celery (*Apium graveolens*) was cultivated in the greenhouse and radish (*Raphanus sativus*) in the adjacent open field. From April through September 2025, pepper (*Capsicum annuum*) was cultivated in both environments. Both sites received supplemental sprinkler irrigation, with timing and frequency adjusted according to crop growth and soil moisture under local production practices; the open field additionally received natural precipitation, whereas the plastic-covered greenhouse received little direct rainfall. Organic fertilizer was the principal fertilizer input in both environments and was applied according to crop growth stage and local practice. Because this was an observational study under commercial production conditions, irrigation volumes and fertilizer application rates were not experimentally standardized; differences in water and fertilizer management were therefore considered potential confounding factors in interpreting the results.

Soil was sampled once per month from October 2024 to September 2025. To reduce transient effects of short-term weather fluctuations, particularly rapid changes in surface soil moisture following rainfall, sampling was scheduled, whenever practicable, after at least three consecutive days without appreciable rainfall and was avoided during rainfall and the immediate post-rainfall period. Greenhouse and open-field soils were sampled on the same day and within similar time windows. Topsoil (0–20 cm) was collected using an S-shaped sampling design. At each sampling event, five randomly collected subsamples were thoroughly mixed to form one composite sample, and three independent composite samples were obtained from each environment. A total of 72 soil samples were collected (12 months × 2 environments × 3 replicates). Visible plant residues, roots, and stones were removed, and homogenized samples were transferred to sterile sealed bags, kept cold immediately after collection, and subsequently stored at −80 °C until DNA extraction.

To characterize both continuous month-to-month dynamics and broader seasonal patterns, the data were analyzed at monthly and seasonal temporal scales. Month-resolved analyses retained the actual sequence of the 12 sampling months from October 2024 to September 2025. Seasons were defined as spring (March–May; Spr), summer (June–August; Sum), autumn (September–November; Aut), and winter (December–February; Win). Seasonal grouping was used to summarize broad annual patterns, whereas continuous temporal dynamics were evaluated using the month-resolved analyses.

Automatic air-temperature and relative-humidity loggers were installed 1.5 m above ground at the center of the greenhouse and at the adjacent open-field site, and measurements were recorded hourly from October 2024 through September 2025. Soil temperature and soil volumetric water content (VWC) sensors were installed only within the 0–20 cm cultivated layer of the greenhouse and operated over the same period. Indoor and outdoor air temperature and relative humidity were used to characterize atmospheric microclimate differences between the two cultivation environments. Greenhouse soil temperature and VWC were used solely to characterize temporal thermal-moisture dynamics within greenhouse soil. Because soil temperature and VWC were not monitored concurrently in the open field, no direct comparison of soil thermal-moisture conditions between greenhouse and open-field soils was made.

### 2.2 DNA extraction, 16S rRNA gene sequencing, and diversity analysis

Soil DNA was extracted using the DNeasy PowerSoil Pro Kit (QIAGEN, United States) according to the manufacturer’s instructions. DNA concentration and purity were assessed using a NanoDrop 2000 spectrophotometer and DNA was stored at −20 °C until analysis. The V3-V4 region of the 16S rRNA gene was amplified by PCR using primers 338F (5’-ACTCCTACGGGAGGCAGCAG-3′) and 806R (5’-GGACTACHVGGGTWTCTAAT-3′), with a barcode incorporated into the forward primer. Amplicons were purified, pooled in equimolar amounts, and sequenced by Majorbio Bio-Pharm Technology Co., Ltd. (Shanghai, China) on an Illumina NovaSeq 6,000 platform using paired-end 250-bp reads (PE250). Raw sequences were quality filtered, merged, and screened for chimeras. Operational taxonomic units (OTUs) were clustered at 97% sequence similarity using UPARSE v7.1 (Edgar, 2013). Sequences assigned to chloroplasts and mitochondria were removed, and all samples were rarefied to the same sequencing depth. Representative OTU sequences were taxonomically annotated against the SILVA 16S rRNA gene database (v138) (Quast et al., 2013). Shannon and Simpson alpha-diversity indices were calculated from the rarefied OTU table.

### 2.3 Potential microbial co-occurrence network construction

Potential bacterial co-occurrence networks were constructed separately for greenhouse and open-field soils. Spearman correlations among OTUs were calculated from the filtered OTU abundance data, and *p* values were adjusted for multiple testing using the false discovery rate (FDR). Isolated nodes without significant connections were removed. The final greenhouse network contained 242 OTU nodes and 481 edges, whereas the open-field network contained 280 OTU nodes and 827 edges. Nodes represented OTUs and edges represented significant positive or negative correlations between OTUs. Network topology was characterized using the number of nodes and edges, average degree, average path length, average clustering coefficient, network density, and modularity. Highly connected OTUs were identified according to node degree and visualized together with their taxonomic assignments.

### 2.4 Statistical analysis

All microbial community analyses were based on the rarefied OTU table. Shannon and Simpson alpha-diversity indices were calculated using the Majorbio Cloud Platform and R. Overall differences in alpha diversity among groups were evaluated using the Kruskal-Wallis H test, followed by Tukey–Kramer *post hoc* comparisons with FDR adjustment of *p* values. Principal coordinate analysis (PCoA) based on Bray-Curtis dissimilarities was used to evaluate differences and temporal variation in bacterial community composition. In addition to the overall comparison between cultivation environments, month-resolved analyses were performed for the 12 actual sampling months. Monthly community centroids for greenhouse and open-field samples were calculated in PCoA space and consecutive months were connected in chronological order (October 2024 to September 2025) to visualize continuous temporal trajectories. Permutational multivariate analysis of variance (PERMANOVA; 9,999 permutations) based on Bray-Curtis dissimilarities was used to test associations of cultivation environment and month with bacterial community composition; pseudo-F, R^2^, and exact *p* values are reported. Linear discriminant analysis effect size (LEfSe) was used to identify differentially abundant taxa (LDA > 2, *p* < 0.05). Redundancy analysis (RDA) was used to evaluate statistical associations between air temperature, relative humidity, and bacterial community structure. Because soil physicochemical properties were not measured concurrently, the RDA was interpreted as an association analysis rather than evidence of a complete community-assembly mechanism or causal environmental driver. Figures were generated using Origin 2024 and the ggplot2 package in R.

## 3 Results

### 3.1 Air temperature and humidity differences and greenhouse soil thermal-moisture dynamics

#### 3.1.1 Hourly-scale temperature and humidity differences

Figure 1A shows the hourly air-temperature difference between the greenhouse and open field (ΔT = greenhouse air temperature—open-field air temperature) from October 2024 to September 2025. Across the year, ΔT ranged from −6.1 to 6.1 °C, revealing pronounced diurnal and seasonal variation in near-surface air temperature differences between the two environments.

**Figure 1:**
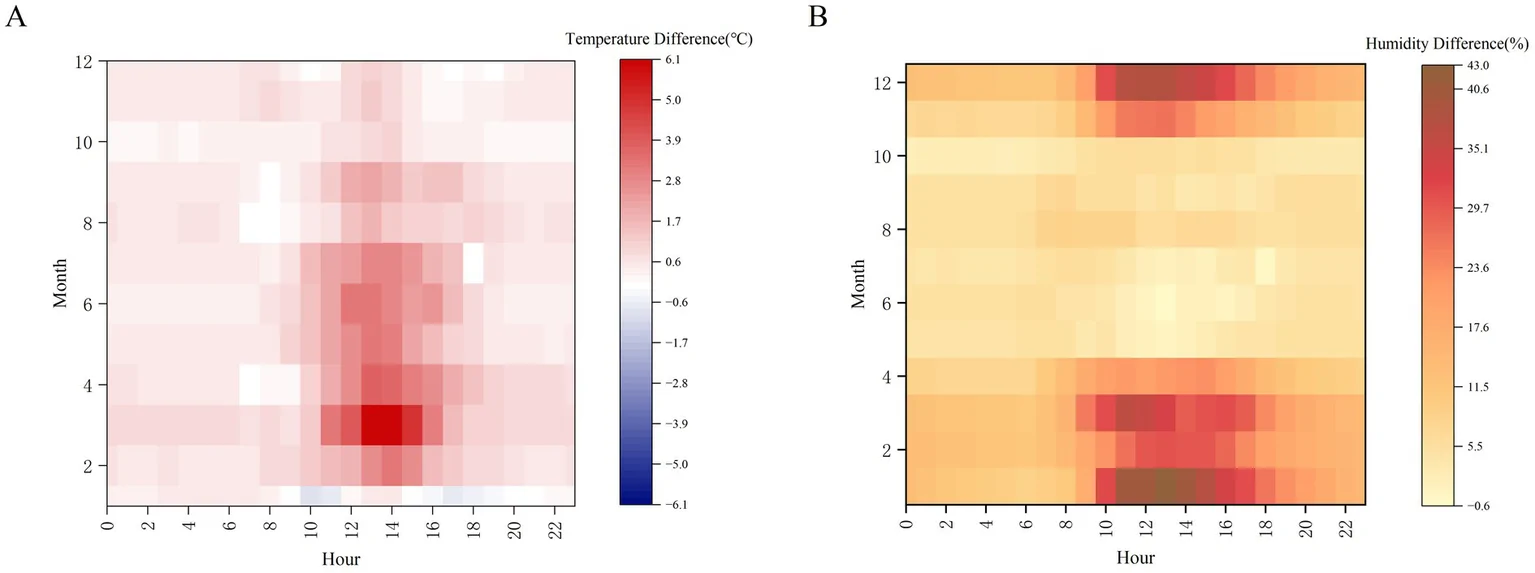
Hourly differences in air temperature and relative humidity between greenhouse and open-field environments. **(A)** Heatmap of hourly air-temperature differences from October 2024 to September 2025 (ΔT = greenhouse air temperature—open-field air temperature). Red indicates higher temperature in the greenhouse and blue indicates lower temperature; color intensity represents the magnitude of the difference. **(B)** Corresponding heatmap of hourly relative-humidity differences (ΔRH = greenhouse RH—open-field RH).

Across the diurnal cycle, the largest temperature differences occurred mainly between 10:00 and 18:00, when ΔT reached a maximum of +6.1 °C, indicating the strongest daytime warming inside the greenhouse. During other periods, ΔT was generally positive but smaller in magnitude, suggesting that the greenhouse also retained heat at night, although the effect was weaker than daytime warming.

Seasonally, the largest positive temperature differences occurred from February to April between 11:00 and 18:00, when ΔT generally exceeded 4 °C and reached a maximum of +6.1 °C. Daytime warming was therefore most pronounced in early spring. Daytime ΔT was also positive from June to August, although the magnitude was slightly lower than that observed from February to April. A brief reversal was observed in January between 17:00 and 19:00, when ΔT fell to approximately −0.6 to −1.7 °C.

Figure 1B shows the hourly difference in relative humidity between the greenhouse and open field (ΔRH = greenhouse RH—open-field RH) over the same period. ΔRH was positive during most hours of the year and ranged from −0.6% to +43.0%, indicating an overall moisture-retention effect of greenhouse covering. The magnitude of ΔRH varied diurnally and seasonally: daytime differences were generally greater than nighttime differences, winter showed the largest differences, summer the smallest, and spring and autumn were intermediate.

During the daytime (10:00–17:00), ΔRH was generally greater than at night and reached 30–40% at its peak. At night (22:00–06:00), ΔRH was lower but remained predominantly positive. During periods of strong midday ventilation in summer, ΔRH approached zero and was occasionally negative, indicating a substantial reduction in the humidity contrast between greenhouse and open-field air.

Seasonally, ΔRH was largest in winter (December–February) and smallest in summer (June–August), with intermediate values in spring and autumn. This seasonal pattern suggests that greenhouse-associated humidity differences may also be modulated by seasonal management practices such as ventilation, shading, and heat conservation.

#### 3.1.2 Seasonal atmospheric conditions and greenhouse soil thermal-moisture dynamics

As shown in Figures 2A,B, greenhouse and open-field air temperature and relative humidity, together with greenhouse soil temperature and VWC, exhibited clear seasonal variation. Greenhouse soil temperature followed a seasonal pattern similar to that of air temperature but varied more smoothly, while greenhouse soil VWC remained within approximately 15–20% throughout the year. Greenhouse air temperature, open-field air temperature, and greenhouse soil temperature all showed unimodal seasonal patterns, with maxima in summer and minima in winter. In spring, summer, and autumn, the order was greenhouse air temperature > greenhouse soil temperature > open-field air temperature; in winter, the order was greenhouse soil temperature > greenhouse air temperature > open-field air temperature. Summer greenhouse air temperature reached 28.5 °C, 1.0 °C above the open field. In winter, greenhouse and open-field air temperatures decreased to 7.5 and 7.0 °C, respectively, whereas greenhouse soil temperature remained at 10.5 °C. Relative humidity in both environments was higher in winter and lower in summer, and greenhouse RH was generally higher than open-field RH. Greenhouse soil VWC was 18%, 20%, 17%, and 15% in spring, summer, autumn, and winter, respectively. The coexistence of high winter air humidity and comparatively low greenhouse soil VWC indicates that atmospheric humidity accumulation and soil water status were not fully synchronized, potentially reflecting irrigation, ventilation, and heat-conservation practices.

**Figure 2:**
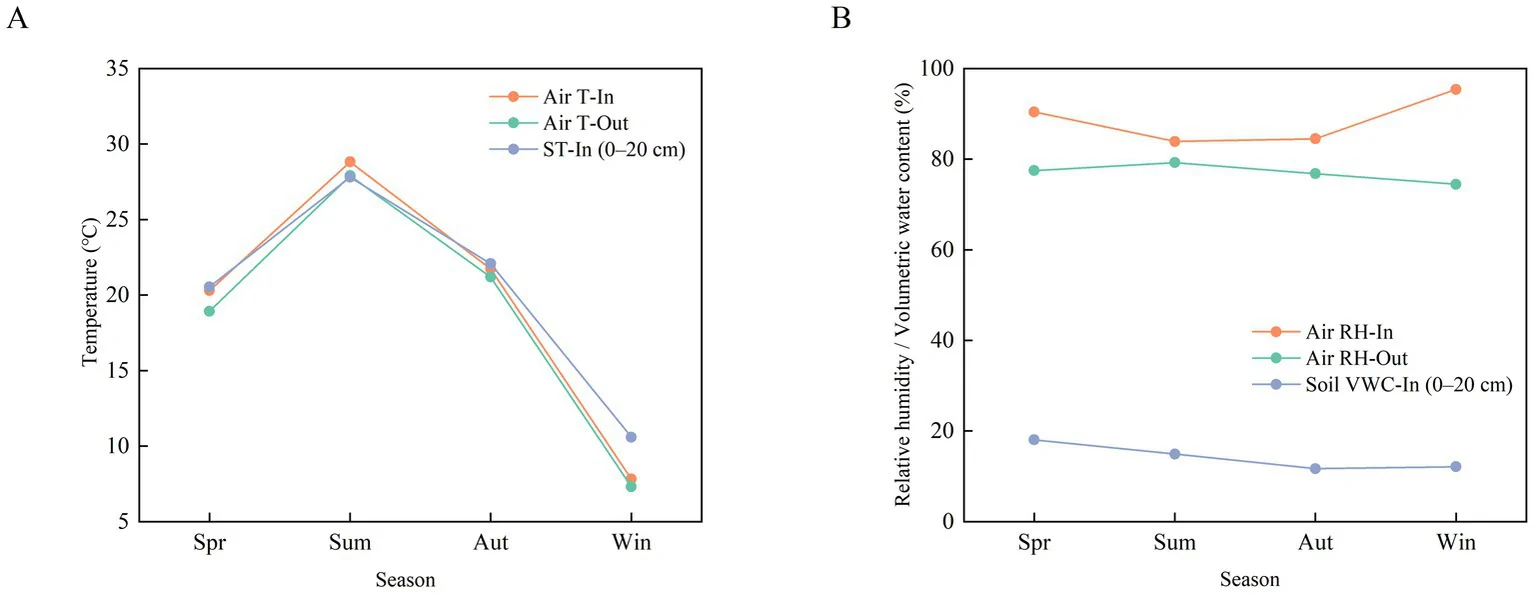
Air temperature, relative humidity, and greenhouse soil thermal-moisture dynamics. **(A)** Seasonal variation in greenhouse air temperature, open-field air temperature, and greenhouse soil temperature. **(B)** Seasonal variation in greenhouse air relative humidity, open-field air relative humidity, and greenhouse soil volumetric water content.

### 3.2 Soil bacterial community composition

At the phylum level (Figure 3A), the dominant taxa were Pseudomonadota, Acidobacteriota, Actinobacteriota, Chloroflexi, and Bacteroidota, together accounting for more than 90% of total bacterial relative abundance. LEfSe identified differentially abundant taxa between greenhouse and open-field soils (LDA > 2, *p* < 0.05). Pseudomonadota, Bacteroidota, and Chloroflexi were relatively more abundant in greenhouse soil, whereas Acidobacteriota and Actinobacteriota were more abundant in open-field soil. These patterns demonstrate differences in bacterial taxonomic composition between cultivation environments, although the environmental factors underlying these differences require evaluation together with soil physicochemical data. At the lowest reliable taxonomic level (Figure 3B), dominant greenhouse-associated taxa included Vicinamibacterales, Sphingomonas, Vicinamibacteraceae, Pyrinomonadaceae, and Pseudarthrobacter, whereas the open-field community was dominated by Sphingomonas, Vicinamibacterales, Pyrinomonadaceae, Pseudarthrobacter, and Vicinamibacteraceae.

**Figure 3:**
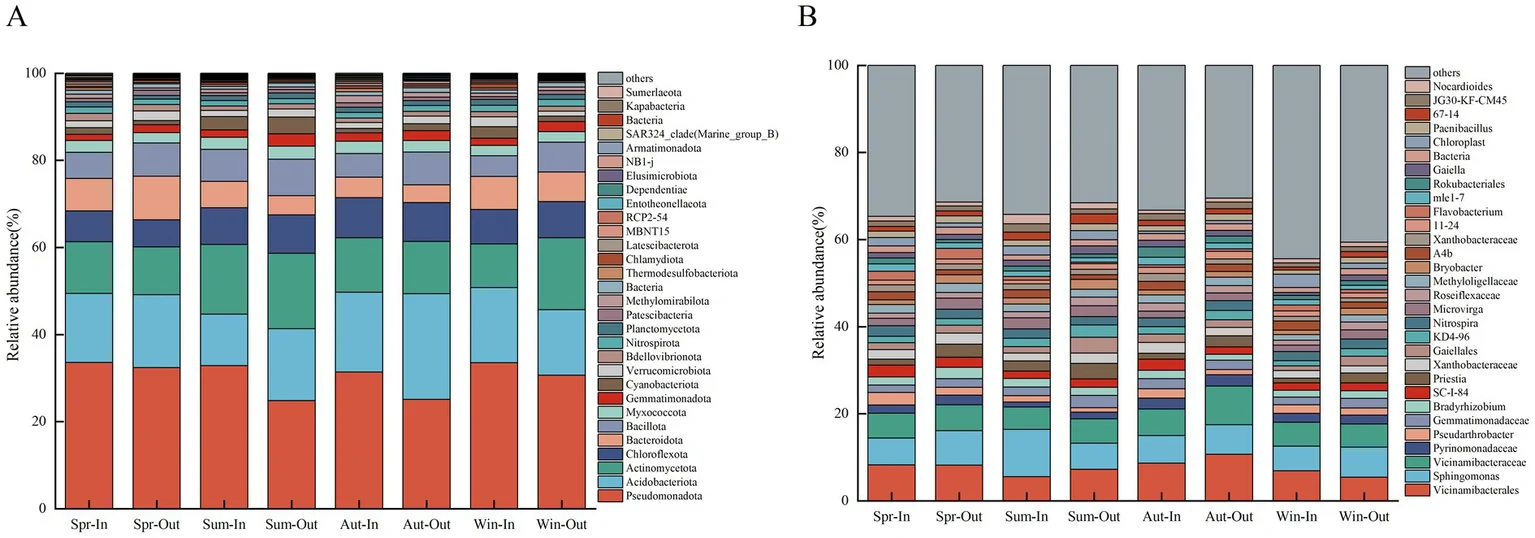
Composition and relative abundance of soil bacterial communities in greenhouse and open-field soils. **(A)** Community composition at the phylum level. **(B)** Community composition at the lowest reliable taxonomic level. Spr, spring; Sum, summer; Aut, autumn; Win, winter; In, greenhouse; Out, open field.

### 3.3 Seasonal variation in soil bacterial alpha diversity

Both Shannon and Simpson indices showed pronounced seasonal variation in bacterial alpha diversity between greenhouse and open-field soils (Figure 4). The Shannon index was significantly higher in greenhouse soil in spring, autumn, and winter but significantly lower in summer; the Simpson index showed the opposite pattern. Environmental differences were strongest in autumn (*p* < 0.01), while differences in the remaining seasons were significant at *p* < 0.05. Thus, alpha-diversity differences between greenhouse and open-field soils were strongly season dependent.

**Figure 4:**
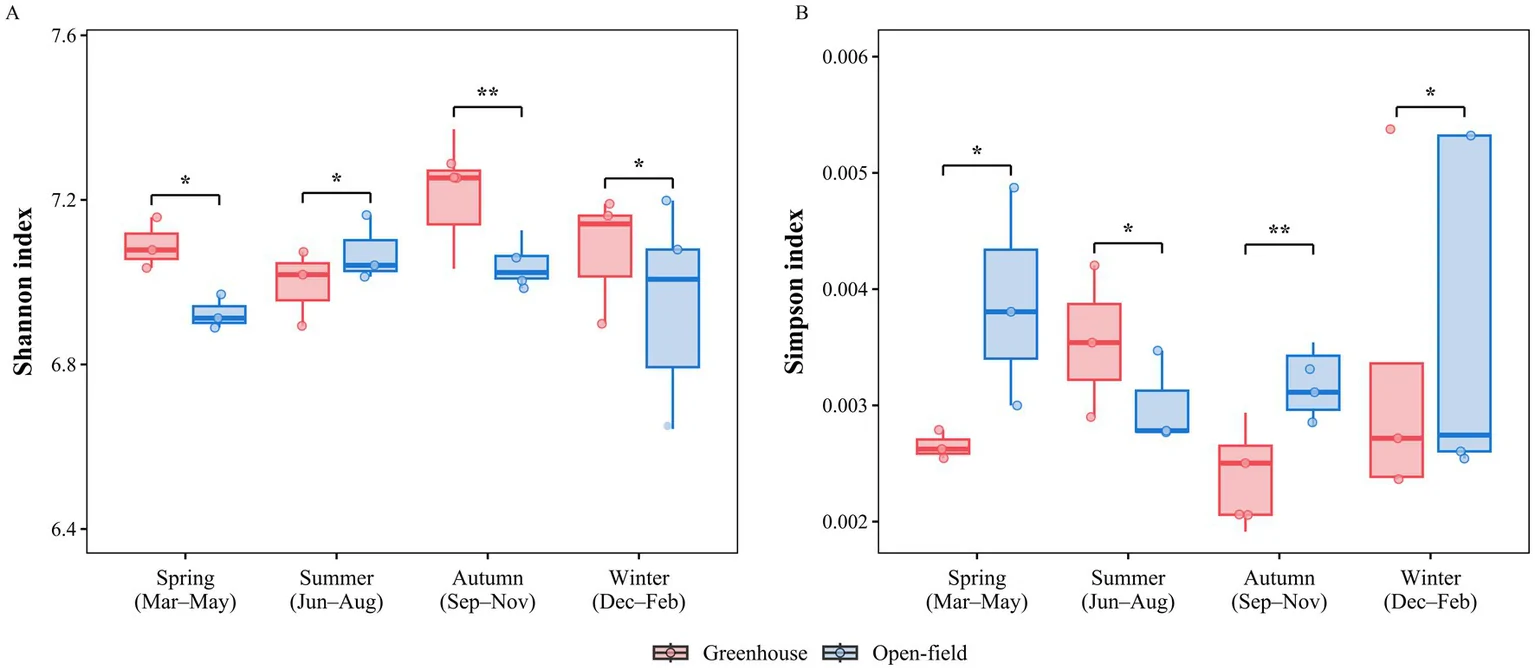
Seasonal variation in alpha diversity of soil bacterial communities in greenhouse and open-field soils. Shannon and Simpson indices are shown for spring (Spr, March–May), summer (Sum, June–August), autumn (Aut, September–November), and winter (Win, December–February) in greenhouse (In) and open-field (Out) soils. Boxplots show group distributions and points represent independent samples. Overall differences were evaluated using the Kruskal-Wallis H test followed by Tukey–Kramer *post hoc* comparisons with FDR-adjusted *p* values. Brackets and asterisks indicate significant greenhouse-open-field differences within the same season: *p < 0.05; **p < 0.01.

### 3.4 Soil bacterial beta diversity and community differentiation

PCoA based on Bray-Curtis dissimilarities showed clear separation of greenhouse and open-field bacterial communities in ordination space (Figure 5A), with PC1 and PC2 explaining 35.79 and 21.02% of community variation, respectively. PERMANOVA further indicated a significant association between cultivation environment and bacterial community composition (pseudo-*F* = 7.32, *R*^2^ = 0.577, *p* < 0.001).

**Figure 5:**
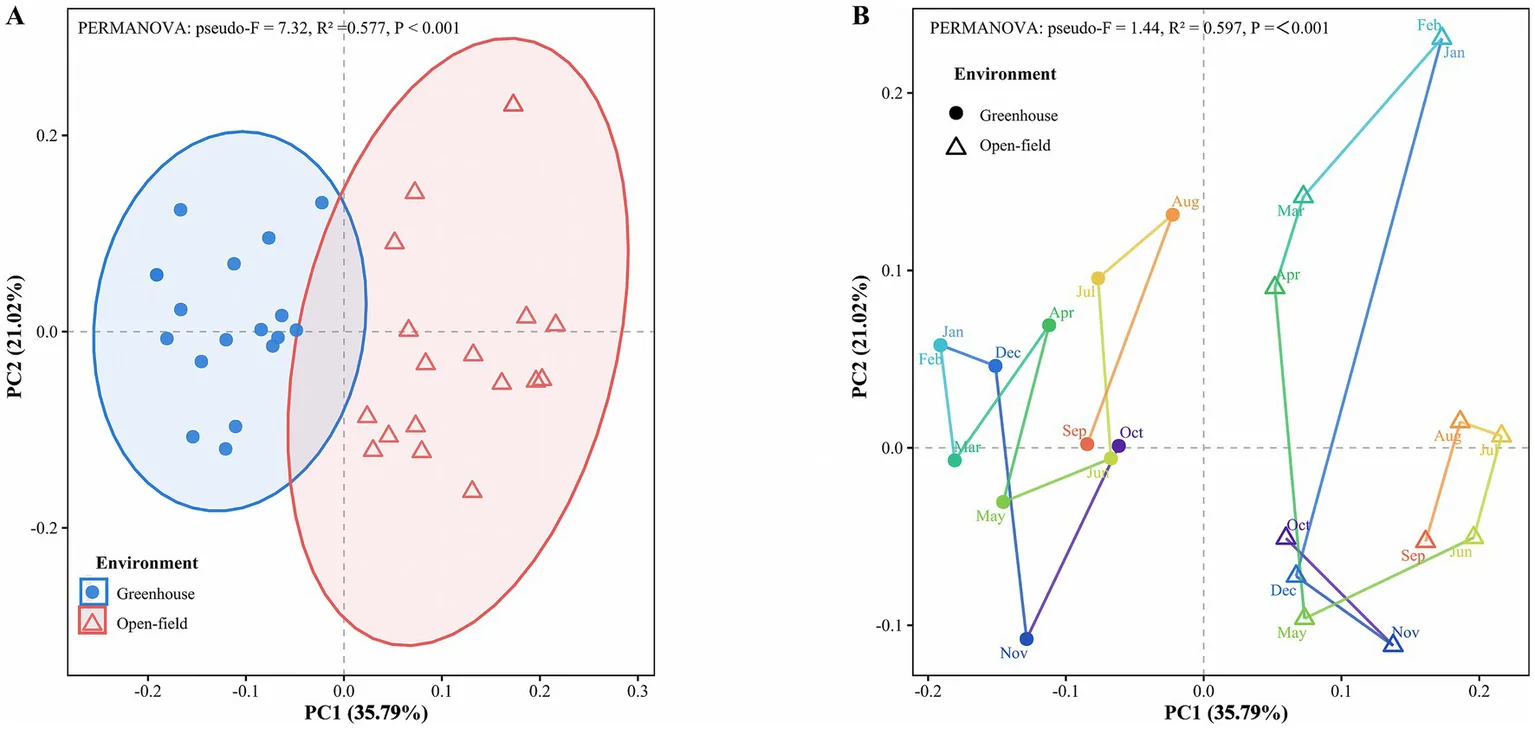
Beta diversity and month-resolved dynamics of soil bacterial communities in greenhouse and open-field soils. **(A)** Principal coordinate analysis (PCoA) based on Bray-Curtis dissimilarities; PC1 and PC2 explain 35.79 and 21.02% of community variation, respectively. Community composition differed significantly between greenhouse and open-field soils (PERMANOVA: pseudo-*F* = 7.32, *R*^2^ = 0.577, *p* < 0.001). **(B)** Month-resolved trajectories of soil bacterial communities across 12 consecutive months. Different symbols represent greenhouse and open-field samples, and colors represent sampling months. Month was significantly associated with community composition (PERMANOVA: pseudo-*F* = 1.44, *R*^2^ = 0.597, *p* < 0.001).

Month-resolved analysis showed clear temporal trajectories in both greenhouse and open-field bacterial communities across the 12-month monitoring period (Figure 5B). PERMANOVA indicated significant differences in community composition among months (pseudo-*F* = 1.44, *R*^2^ = 0.597, *p* < 0.001), demonstrating pronounced month-to-month dynamics. This analysis retained temporal information that was not fully represented by seasonal grouping.

### 3.5 Relationships between air temperature, relative humidity, and bacterial community structure

RDA was used to evaluate statistical associations of air temperature (Temp) and relative humidity (RH) with soil bacterial community structure. RDA1 and RDA2 explained 9.03 and 6.56% of community variation, respectively, for a cumulative explained variance of 15.59% (Figure 6). The measured atmospheric microclimate variables therefore accounted for only a limited fraction of variation in bacterial community structure.

**Figure 6:**
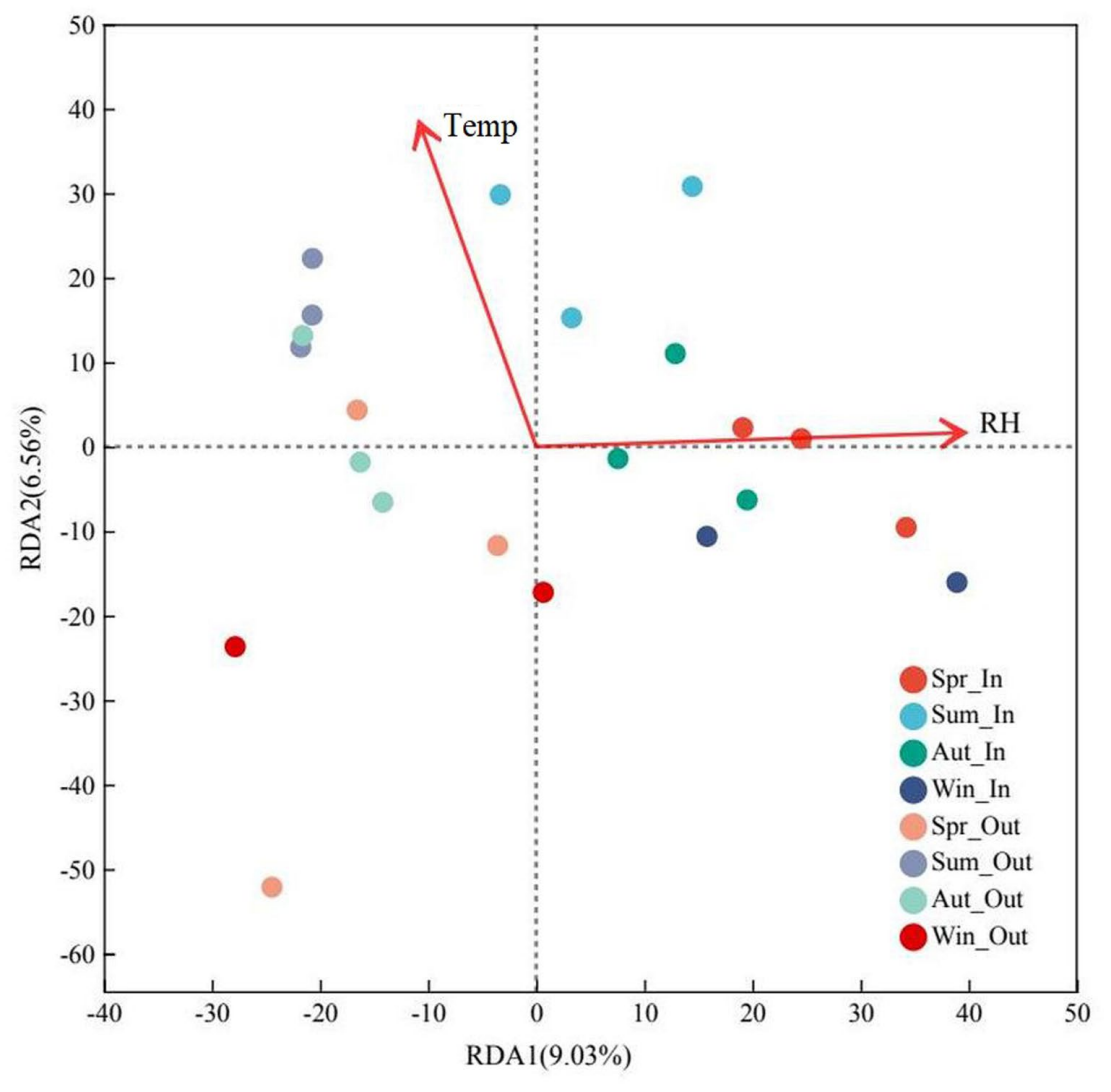
Redundancy analysis (RDA) showing statistical associations of air temperature (Temp) and relative humidity (RH) with soil bacterial community structure. RDA1 and RDA2 explain 9.03 and 6.56% of community variation, respectively, for a cumulative explained variance of 15.59%. Because soil physicochemical properties were not measured concurrently, this analysis is used only to evaluate statistical associations between the measured atmospheric microclimate variables and bacterial community structure and not to identify the principal drivers of community change. Spr, spring; Sum, summer; Aut, autumn; Win, winter; In, greenhouse; Out, open field.

In the RDA ordination, samples showed partial separation by environment and season. The air-temperature vector was oriented mainly toward negative RDA1 and positive RDA2, whereas the relative-humidity vector extended primarily along positive RDA1, indicating different directions of statistical association with community structure. Seasonal samples were also dispersed in ordination space, consistent with temporal covariation between bacterial community composition and atmospheric temperature and relative humidity.

Most variation in bacterial community structure remained unexplained by the measured air temperature and relative humidity. Because soil pH, electrical conductivity, organic matter, salinity, available nutrients, and other soil physicochemical properties that can be closely related to microbial communities were not measured concurrently, the RDA should be interpreted as describing statistical associations between the measured atmospheric microclimate variables and bacterial community structure, rather than identifying the principal drivers of community change or establishing causality.

### 3.6 Potential bacterial co-occurrence network characteristics

Potential bacterial co-occurrence networks were constructed separately for greenhouse and open-field soils (Figure 7), and the main topological properties are summarized in Table 1. The greenhouse network contained 242 nodes and 481 edges, whereas the open-field network contained 280 nodes and 827 edges. Compared with the greenhouse network, the open-field network had a higher average degree (5.907 vs. 3.975), average clustering coefficient (0.381 vs. 0.324), and network density (0.021 vs. 0.016), as well as a shorter average path length (5.367 vs. 5.749). Modularity was slightly higher in the greenhouse network than in the open-field network (0.699 vs. 0.683). Under the network-construction criteria used here, the open-field community therefore exhibited a denser potential association structure.

**Figure 7:**
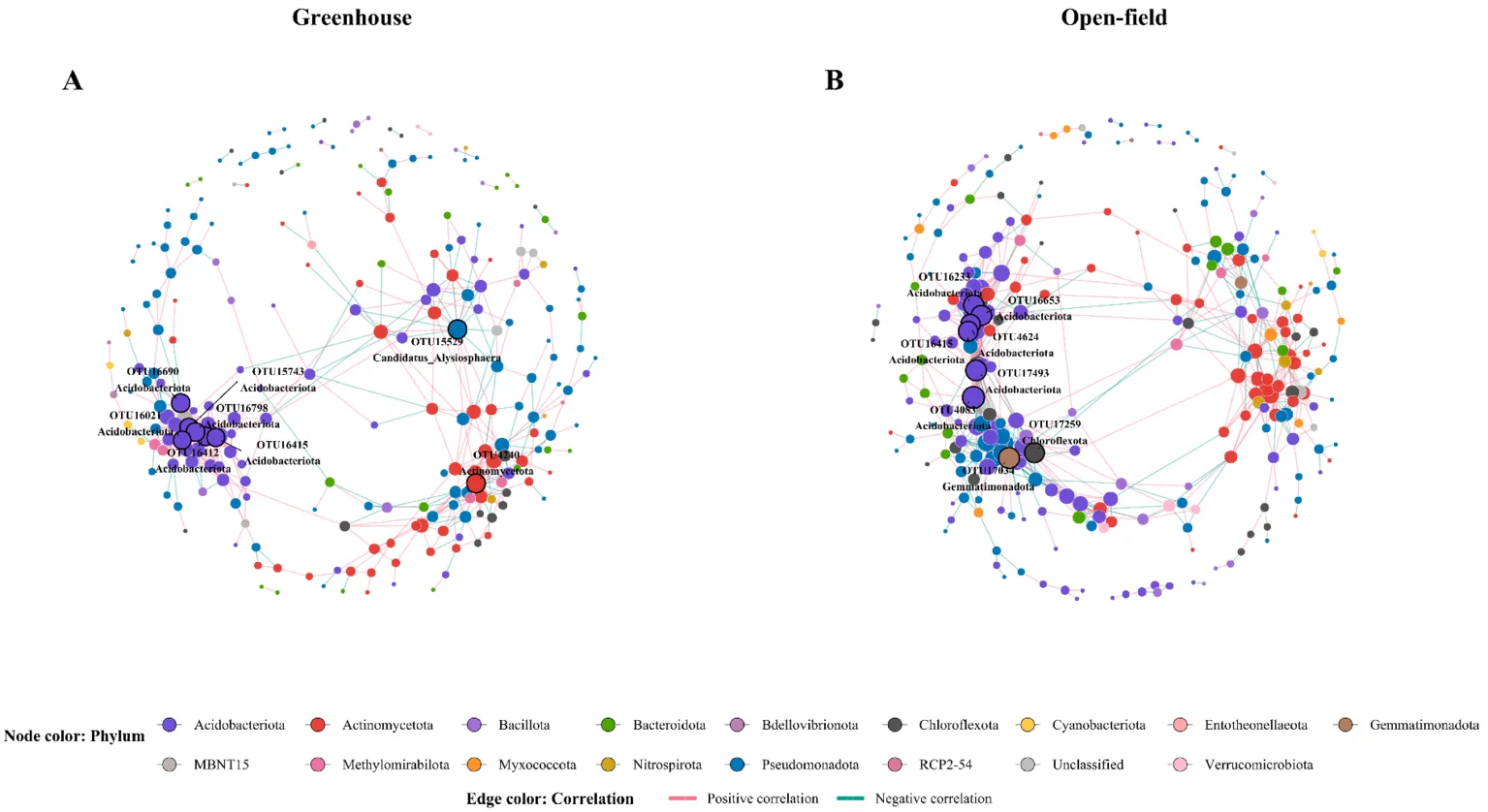
Potential bacterial co-occurrence networks in greenhouse and open-field soils. **(A)** Greenhouse; **(B)** open field. Nodes represent bacterial OTUs; node colors indicate phylum-level taxonomy and node size is proportional to degree. Edges represent significant Spearman correlations meeting the network-screening criteria; red edges indicate positive correlations and green edges indicate negative correlations.

**Table 1:** Topological properties of potential soil bacterial co-occurrence networks.

Environment	Nodes	Edges	Average degree	Average path length	Average clustering coefficient	Network density	Negative edges (%)	Modularity
Greenhouse	242	481	3.975	5.749	0.324	0.016	24.32	0.699
Open field	280	827	5.907	5.367	0.381	0.021	33.01	0.683

Nodes represent bacterial OTUs, and edges represent significant correlations retained under the network-construction criteria.

Positive correlations predominated in both networks. Positive edges accounted for 75.68% of greenhouse edges and 66.99% of open-field edges, whereas negative edges accounted for 24.32 and 33.01%, respectively. Thus, the greenhouse network had a higher proportion of positive correlations, whereas negative correlations were relatively more frequent in the open field, indicating differences in statistical co-variation patterns among bacterial OTUs.

Eight highly connected OTUs were identified in each network based on node degree and were annotated with OTU identifiers and taxonomic information in Figure 7. The composition of these highly connected OTUs differed between the two environments, indicating that different bacterial taxa occupied highly connected network positions under greenhouse and open-field cultivation.

## 4 Discussion

This study revealed clear differences between greenhouse and open-field cultivation environments in near-surface atmospheric microclimate, soil bacterial community composition, and potential co-occurrence network characteristics, while bacterial communities also exhibited significant temporal dynamics throughout the 12-month monitoring period. RDA further indicated that the measured air temperature and relative humidity were statistically associated with bacterial community structure, although their explanatory power was limited. Therefore, our findings primarily reflect associations between greenhouse-associated microclimatic conditions and changes in bacterial communities, rather than demonstrating that atmospheric temperature and humidity are direct or predominant drivers of bacterial community differentiation between greenhouse and open-field environments. Other factors not included in the current RDA, including soil physicochemical properties, crop development, and agricultural management practices, may have jointly contributed to the observed differences in bacterial communities.

### 4.1 Greenhouse-associated regulation of temperature and humidity dynamics

The greenhouse environment was characterized by daytime warming, nighttime heat retention, and persistently higher relative humidity, with pronounced diurnal and seasonal dynamics (Rabbi et al., 2019; Yang et al., 2024). During the day, solar radiation transmitted through the plastic film promoted heat accumulation, and the warming effect was strongest in early spring, when the maximum temperature difference reached +6.1 °C. The brief negative temperature difference around sunset in winter may reflect transient greenhouse heat loss. Higher greenhouse relative humidity is consistent with crop transpiration and soil evaporation combined with restricted water-vapor exchange under plastic covering. In summer, ventilation and shading likely reduced humidity differences between greenhouse and open-field environments, consistent with measurements showing strong effects of ventilation on greenhouse temperature and humidity distributions (Mao et al., 2024). Greenhouse soil temperature and VWC also showed relatively smooth seasonal variation. Importantly, because corresponding soil temperature and VWC were not monitored in the open field, these data describe temporal stability within greenhouse soil and do not demonstrate stronger soil buffering relative to open-field soil.

### 4.2 Environmental and temporal differences in soil bacterial community composition and diversity

Bacterial community composition differed substantially between greenhouse and open-field soils. At the phylum level, Pseudomonadota, Bacteroidota, and Chloroflexi were relatively more abundant in greenhouse soil, whereas Acidobacteriota and Actinobacteriota were more abundant in open-field soil. At the lowest reliable taxonomic level, Sphingomonas, Vicinamibacterales, and Vicinamibacteraceae were relatively more abundant in greenhouse soil, whereas Pyrinomonadaceae and Pseudarthrobacter were more abundant in open-field soil. These differences demonstrate environmental differentiation in bacterial taxonomic composition. Changes in relative abundance may reflect distinct responses to local environmental conditions; previous studies have shown that soil moisture, salinity, and nutrient availability can coincide with changes in dominant bacterial taxa. However, 16S rRNA gene amplicon data alone do not establish the specific ecological preferences or physiological mechanisms of these taxa.

Shannon and Simpson indices varied seasonally, and the direction of greenhouse-open-field differences changed among seasons. Thus, the relationship between cultivation environment and bacterial alpha diversity was time dependent rather than consistently positive or negative throughout the year. Comparable temporal variation has been reported in other soil microbial studies (Koranda et al., 2013; Li et al., 2022; Bon et al., 2026), including subtropical systems in which bacterial communities and co-occurrence networks differed between wet and dry seasons (Lin et al., 2023). Warming experiments have also shown that bacterial responses can depend on crop type and can be reflected in alpha diversity and highly connected network taxa (Zhong et al., 2024). Evaluations of greenhouse effects on soil bacterial diversity should therefore account for sampling time and seasonal context.

Month-resolved analysis further revealed continuous temporal dynamics that could not be fully captured by grouping samples into four seasons. Month was significantly associated with bacterial community composition (PERMANOVA, *R*^2^ = 0.597, *p* < 0.001), and both greenhouse and open-field communities followed continuous trajectories through the 12-month monitoring period. Combining seasonal alpha-diversity analysis with month-resolved beta-diversity analysis therefore provides complementary perspectives on annual bacterial community dynamics.

### 4.3 Associations between atmospheric temperature, humidity, and bacterial community differentiation

Air temperature and relative humidity jointly explained 15.59% of the variation in soil bacterial community structure, indicating a detectable but limited statistical association between the measured atmospheric microclimate and bacterial communities. Soil bacteria inhabit the soil microenvironment directly, and their community structure is also related to edaphic conditions. Greenhouse soil pH and electrical conductivity have been identified as key factors associated with bacterial diversity and composition (Kim et al., 2016), while long-term greenhouse monoculture and salt accumulation can coincide with changes in soil properties and rhizobacterial communities (Hahm et al., 2017). Long-term plastic greenhouse cultivation can also be accompanied by soil acidification, nutrient accumulation, and bacterial community shifts (Song et al., 2018), and large-scale comparisons of greenhouse and open-field cultivation have demonstrated associations of multiple soil properties and contaminants with microbial diversity (Sun et al., 2018). Recent water and fertilizer studies further identify soil water content, pH, nitrogen forms, electrical conductivity, available potassium, and plant biomass as important correlates of bacterial community composition (Ning et al., 2024; Shen et al., 2024). Consequently, the greenhouse-open-field bacterial community differences observed here cannot be attributed specifically to atmospheric temperature and humidity.

### 4.4 Differences in potential bacterial co-occurrence networks under greenhouse and open-field cultivation

The potential co-occurrence networks also differed between greenhouse and open-field soils. The open-field network contained more nodes and edges and had higher average degree, clustering coefficient, and density, together with a shorter average path length. Under the network-construction criteria used in this study, these properties indicate a denser pattern of potential associations among open-field bacterial OTUs. In contrast, modularity was slightly higher in the greenhouse network (0.699 vs. 0.683), illustrating that network connectivity and modular organization do not necessarily change in parallel. A single topological metric should therefore not be used to infer that one bacterial network is intrinsically more complex or stable than another. Changes in microbial co-occurrence networks have also been reported under long-term greenhouse cultivation, irrigation treatments, seasonal variation, and experimental warming (Dang et al., 2023; Lin et al., 2023).

Both networks were dominated by positive correlations, but the proportions of positive and negative edges differed. Positive edges represented 75.68% of the greenhouse network and 66.99% of the open-field network, whereas negative edges accounted for 24.32 and 33.01%, respectively. These patterns describe differences in statistical co-variation among OTU abundances. Co-occurrence correlations, however, should not be equated with direct ecological interactions: positive correlations do not necessarily indicate facilitation or cooperation, and negative correlations do not necessarily indicate competition or inhibition. Functional measurements, resource-use information, and process-rate data would be required to clarify their ecological meaning.

The composition of highly connected OTUs also differed between the two networks. Although these taxa occupied prominent topological positions, high node degree alone does not establish a keystone ecological function. We therefore describe them conservatively as highly connected OTUs rather than as keystone taxa. The observed network differences likewise cannot be attributed solely to greenhouse-associated temperature and humidity changes because soil pH, electrical conductivity, organic matter, available nutrients, crop growth, irrigation, and fertilization can all influence bacterial communities and their co-occurrence patterns (Ning et al., 2024; Shen et al., 2024).

### 4.5 Limitations and future perspectives

This year-round observational study systematically compared atmospheric microclimate and soil bacterial community dynamics between greenhouse and open-field cultivation environments, but several limitations constrain mechanistic interpretation. First, soil temperature and VWC were monitored only within the greenhouse. We therefore could not determine whether soil thermal-moisture regimes differed between greenhouse and open-field soils or quantitatively attribute between-environment bacterial community differentiation to soil temperature or moisture. Future reciprocal soil-transplant experiments, in which greenhouse and open-field soils are exposed to both greenhouse and outdoor environments, could help separate current microclimate effects from soil legacy and management-history effects. Second, soil physicochemical properties and quantitative water and fertilizer inputs were not monitored concurrently; the observed community differences should therefore be interpreted as ecological responses to the combined conditions of the two cultivation environments rather than to a single measured driver. Third, the study involved one paired greenhouse-open-field production system, so inference should be restricted to this study system rather than generalized to greenhouse cultivation as a whole. Despite these limitations, the combination of continuous one-year microclimate monitoring, month-resolved community analysis, and potential co-occurrence network comparison provides a detailed temporal description of bacterial communities in a subtropical protected-agriculture system. Future studies should monitor soil thermal-moisture conditions and physicochemical properties synchronously in both environments and incorporate quantitative management records or controlled experiments to resolve the relative contributions of environmental factors.

## 5 Conclusion

Year-round monitoring showed clear differences in near-surface air temperature and relative humidity between greenhouse and open-field environments, while greenhouse soil temperature and VWC exhibited comparatively smooth temporal variation. Soil bacterial community composition differed significantly between greenhouse and open-field soils, and the direction of alpha-diversity differences varied among seasons. Month-resolved analysis further demonstrated significant temporal dynamics across the 12-month monitoring period. Air temperature and relative humidity were statistically associated with bacterial community structure but explained only 15.59% of total variation, indicating that the measured atmospheric microclimate variables alone were insufficient to account for bacterial community differentiation between cultivation environments. Potential bacterial co-occurrence networks also differed in topology. Overall, the results demonstrate associations among greenhouse-related microclimate conditions, bacterial community composition, and temporal dynamics, but they do not identify the principal causal drivers of community change because concurrent soil physicochemical data were unavailable and crop development and agricultural management represented potential confounding factors. Future studies integrating synchronous soil-environment monitoring, soil physicochemical measurements, and quantitative management records are needed to resolve the environmental mechanisms underlying bacterial community variation in protected cultivation systems.

## Data Availability

The datasets presented in this study can be found in online repositories. The names of the repository/repositories and accession number (s) can be found at: https://www.ncbi.nlm.nih.gov/, PRJNA1476812.
